# Discovery of a novel fibroblast activation protein (FAP) inhibitor, BR103354, with anti-diabetic and anti-steatotic effects

**DOI:** 10.1038/s41598-020-77978-z

**Published:** 2020-12-04

**Authors:** Jae Min Cho, Eun Hee Yang, Wenying Quan, Eun Hye Nam, Hyae Gyeong Cheon

**Affiliations:** 1 Innovative Drug Research Institute, Boryung Pharm. Co., Ltd, Danwon-gu, Ansan-si, Gyeonggi-do 15425 South Korea; 2 Department of Pharmacology, College of Medicine, Gachon University, Incheon, 21999 South Korea

**Keywords:** Drug discovery, Endocrinology, Molecular medicine

## Abstract

Fibroblast growth factor (FGF) 21 is a class of hepatokines that plays a protective role against obesity, insulin resistance, and liver damage. Despite this, protective effects of FGF21 in human appear to be minimal, possibly due to its proteolytic cleavage by the fibroblast activation protein (FAP). Here, we presented a novel FAP inhibitor, BR103354, and described its pharmacological activities as a potential therapeutic agent for the treatment of metabolic disorders. BR103354 inhibited FAP with an IC_50_ value of 14 nM, showing high selectivity against dipeptidyl peptidase (DPP)-related enzymes and prolyl oligopeptidase (PREP). In differentiated 3T3/L1 adipocytes, the addition of FAP diminished hFGF21-induced Glut1 and phosphorylated levels of ERK, which were restored by BR103354. BR103354 exhibited good pharmacokinetic properties as evidenced by oral bioavailability of 48.4% and minimal hERG inhibition. Single co-administration of BR103354 with hFGF21 reduced nonfasting blood glucose concentrations, in association with increased intact form of hFGF21 in ob/ob mice. Additionally, chronic treatment of BR103354 for 4 weeks reduced nonfasting blood glucose concentrations with improved glucose tolerance and with reduced triglyceride (TG) content in liver of ob/ob mice. Consistently, BR103354 improved hepatic steatosis and fibrosis in a choline-deficient, L-amino acid-defined, high-fat diet (CDAHFD)-induced non-alcoholic steatohepatitis (NASH) mouse model. FAP inhibitory effects of BR103354 were confirmed in normal cynomolgus monkeys. Together, BR103354 acts as an effective FAP inhibitor in vitro and in vivo, thereby demonstrating its potential application as an anti-diabetic and anti-NASH agent.

## Introduction

Type 2 diabetes is a major cause of death worldwide, and increasing cases pose a serious threat to human health^1^. The major characteristics of this disease includes insulin resistance in the liver, skeletal muscle, adipose tissue, and brain, and the failure of the pancreatic β-cell to compensate for insulin resistance by increasing insulin secretion^2^. There exists a variety of anti-diabetic agents which reduce insulin resistance (metformin, thiazolidinediones), increase insulin secretion (sulfonylureas, DPP 4 inhibitors, incretin mimetics), or release glucose from the urine (sodium/glucose cotransporter 2 inhibitors)^3^. Nevertheless, new therapeutic agents are needed to reinforce therapeutic effectiveness and to overcome reported side effects.

FGF21 is an endocrine hormone, that belongs to the FGF family and is mainly secreted by the liver, some of which are also secreted in the adipose tissue^4,5^. During fasting, FGF21 produced by peroxisome proliferator-activated receptor-alpha (PPAR-α) is required for activation of free fatty acid (FFA) oxidation, lipolysis, and ketogenesis^6,7^, meaning that FGF21 plays an important role in the adaptation to fasting^4^. Moreover, increased circulating FGF21 levels have been observed in cases of obesity, type 2 diabetes, or nonalcoholic fatty liver disease^8–11^, which suggests its protective effects against various metabolic disorders. Recently, FGF21 was additionally classified as a batokine, secreted from brown adipose tissue, playing an important role in cold-induced thermogenesis via upregulation of UCP1^12^.

FGF21 is known to transmit signals towards a cell’s interior by binding its N-terminus to FGF receptors (FGFRs) and its C-terminus to beta-klotho (β-klotho)^13^. In adipocytes, activation of the receptor leads to translocation of Glut1 to the plasma membrane, thus increasing the cellular glucose uptake^14^, whereas increased hepatic fatty acid oxidation was observed by FGF21^6–9^. Consequently, FGF21 reduces blood glucose and triglyceride levels in diet-induced obese mice^15,16^ and in obese diabetic cynomolgus monkeys^17^. Based on these observations, Eli Lilly developed the FGF21 analog, LY2405319, and confirmed the improvement of metabolic disorders in rodents and nonhuman primates^18^. However, LY2405319 administration to obese human subjects with type 2 diabetes resulted in lower body weight and lipid levels, but did not reduce blood glucose levels^19^.

FAP (also known as FAP-α, or seprase) is a member of the serine protease DPP family of enzymes, consisting of DPP 4, FAP, DPP 2, DPP 8, DPP 9, and PREP, and shares 52% amino acid identity with DPP 4 (also described as CD26 or FAP-β)^20–24^. FAP has dual activities as both a post-proline dipeptidase and an endopeptidase. FAP is an integral membrane protein that exists as a 170-kDa homodimer and is also found in human serum as a soluble form lacking the transmembrane domain. FAP is expressed at sites of tissue remodeling^25^ and also activates the hepatic stellate cells^26^. Recent reports have shown that human FGF21 is subjected to proteolytic cleavage at both the N-terminus and C-terminus by FAP, thereby contributing to its short half-life^27–30^. While the cleavage at the four amino acid residues of N-terminus of FGF21 does not affect its action, Gly-Pro cleavage of FGF21 at the C-terminus by FAP generates inactive FGF21^31,32^. To overcome the limitation of rapid cleavage of hFGF21 by FAP, various approaches have been investigated, such as the attachment of polyethylene glycol to FGF21^33–35^ and the fusion of FGF21 to an Fc fragment^28^, as treatments for diabetes and metabolic diseases.

FAP inhibition, on the other hand, was proposed as a new strategy to enhance FGF21 activity and has proven to be effective in animal models^29,36–38^. In support of this approach, an FAP knockout mouse prevented liver steatosis, insulin resistance, glucose tolerance, and increased FGF21 in diet-induced obese mice^38^. In pursuit of developing a novel and potent FAP inhibitor, we screened a compound library and carried out optimization research, ending with the discovery of BR103354 as a new FAP inhibitor. In the present study, we have reported on the pharmacological and pharmacokinetic properties of BR103354 for evaluation of its potential as an anti-diabetic and anti-NASH agent.

## Results

### FAP inhibition by BR103354

**Figure 1:**
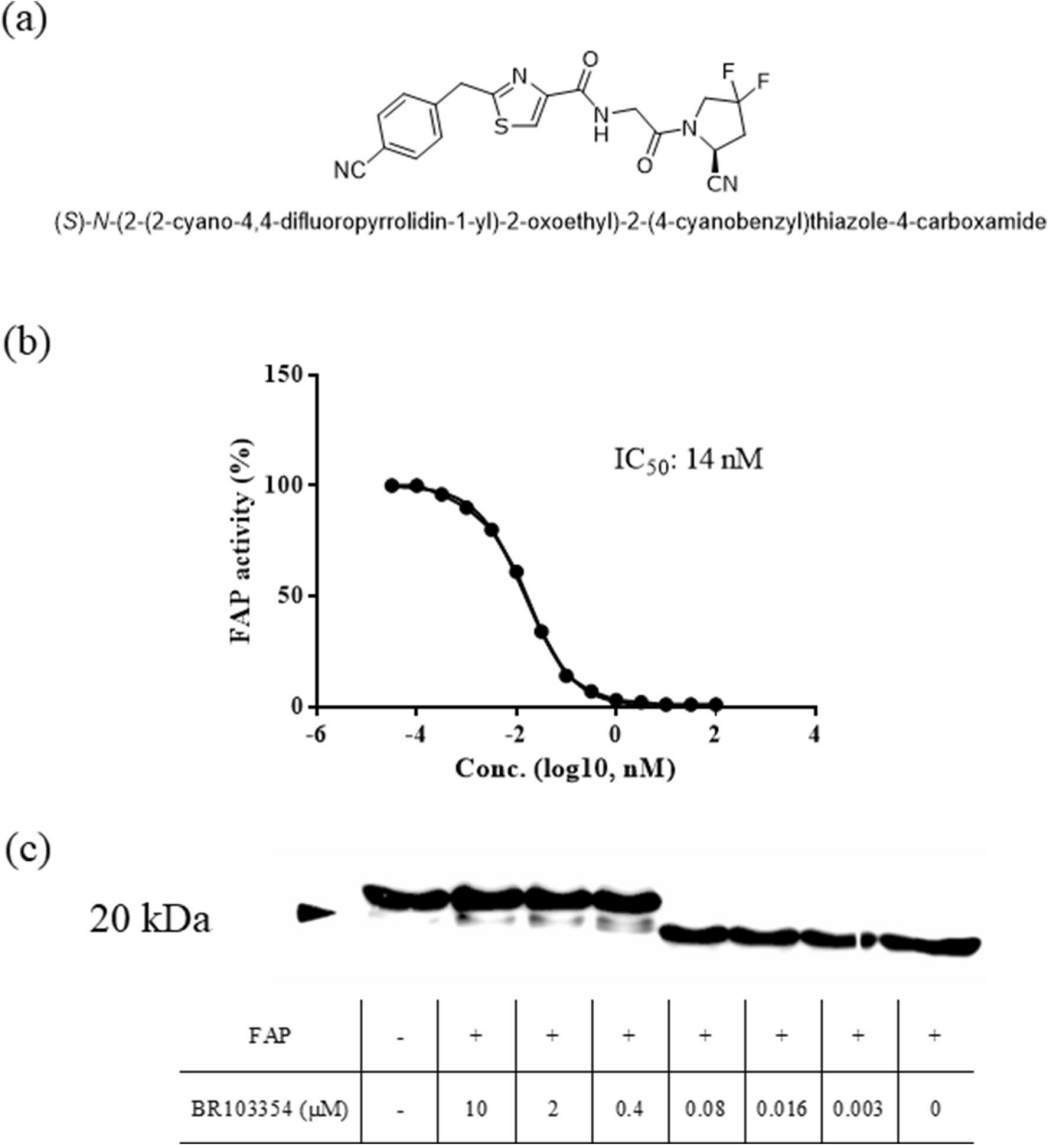
In vitro FAP potency of BR103354. (**a**) Chemical structure of BR103354. (**b**) The concentration-dependent inhibitory effects of BR103354 on FAP activity. A commercially available FAP substrate with the sequence Suc-Gly-Pro-Leu-Gly-Pro-AMC was used for the assay. (**c**) Recombinant human FGF21 (200 nM) was co-incubated with recombinant human FAP (50 nM) and the indicated concentrations of BR103354 in buffer and subjected to immunoblot analysis with anti-hFGF21 (hFGF21:hFAP = 4:1, at a molar ratio). As a control, 0.1% dimethyl sulfoxide (vehicle) was tested.

BR103354 inhibited FAP activity in a concentration-dependent manner with an estimated IC_50_ of 14 nM and showed high selectivity against other related proteases with a selectivity index (SI) of 27.6 against PREP (Table 1, Fig. 1b). When full-length recombinant hFGF21 was incubated with FAP in an assay buffer in a cell-free system, hFGF21 cleavage occurred rapidly, but cleavage was inhibited in the presence of BR103354 starting at a concentration of 0.4 µM, as determined by sodium dodecyl sulphate–polyacrylamide gel electrophoresis (SDS-PAGE) (Fig. 1c), thus indicating that BR103354 was capable of blocking FAP-mediated cleavage of hFGF21.

**Table 1:** In vitro potency and selectivity of BR103354.

MW	clogP	IC_50_ (nM)^a^					SI index^b^ (PREP/FAP)
		FAP	PREP	DPP4	DPP2	DPP9	
415.42	1.907	14	387	> 100,000	n.d	5,036	27.6

^a^The inhibitory potency of BR103354 on FAP and selectivity against DPP family enzymes were determined by fluorometric assay using AMC substrates and presented as IC_50_ values. ^b^SI means selectivity index (calculated as [IC_50_(PREP)/IC_50_(FAP)]); n.d., not detected.

### Effects of BR103354 in differentiated 3T3/L1 adipocytes

**Figure 2:**
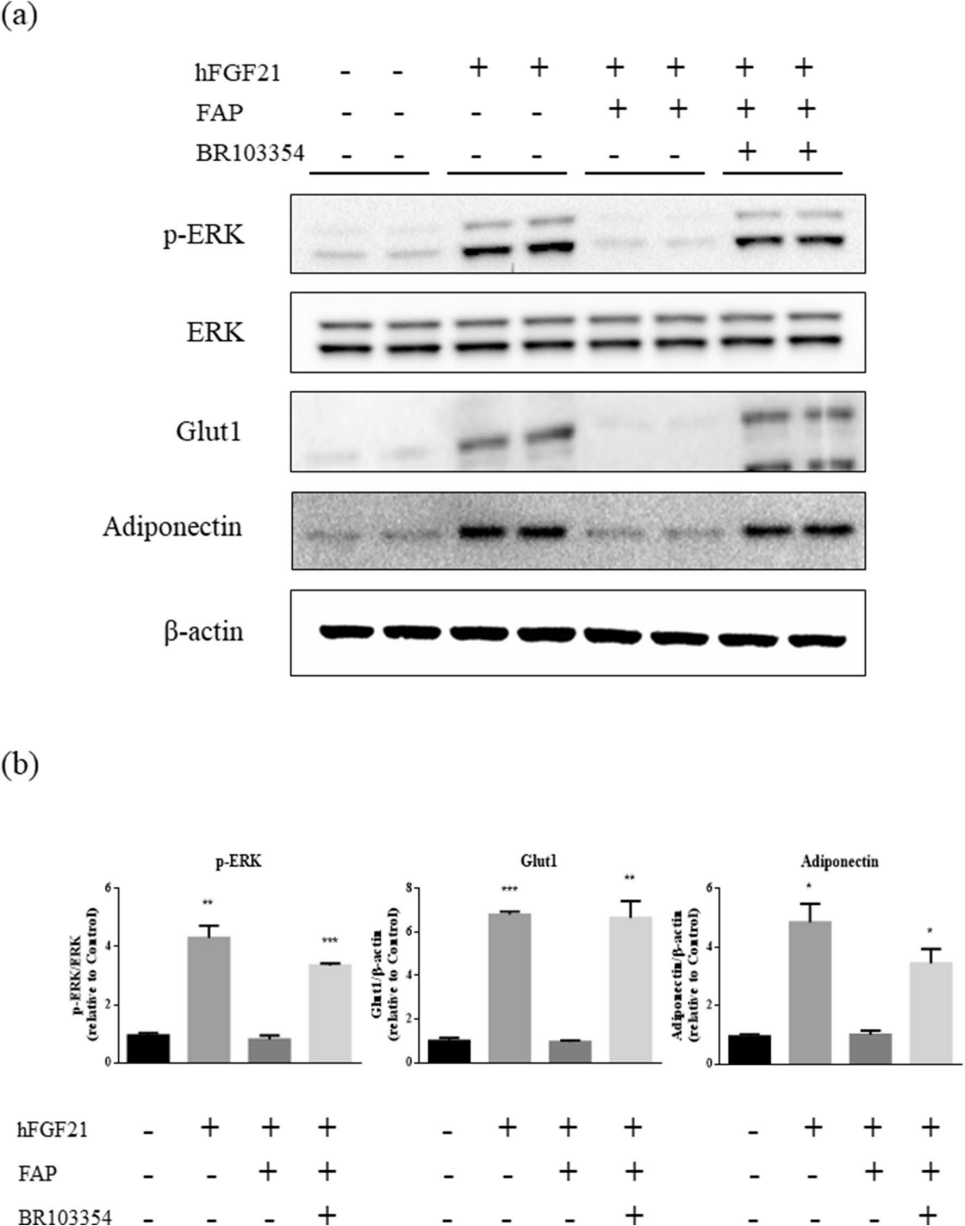
Enhanced ERK signaling in 3T3/L1 cells by BR103354. (**a**) Fully differentiated 3T3/L1 adipocytes were incubated with full-length hFGF21 at 500 ng/mL. FGF21 was pre-incubated with PBS or recombinant FAP at 100 ng/mL (substrate: enzyme ratio = 5:1) for 2 h, with or without 5 µM of BR103354. Phosphorylated ERK (p-ERK), total ERK (ERK), Glut1, adiponectin and β-actin were measured by immunoblots. (**b**) Quantification of p-ERK, Glut1 and adiponectin was obtained with densitometric analysis, and normalized with total ERK or β-actin, respectively. All data are the means ± S.D. *P < 0.05, **P < 0.01 and ***P < 0.001 vs. no treatment (control) group.

Unlike the significant role of FAP in the regulation of endogenous hFGF21 in humans, endogenous mFGF21 levels are not significantly influenced by FAP since the Gly at the C-terminus of FGF21 is substituted with Glu in mice^30^. Thus, to determine the effects of BR103354 on the cellular signaling pathway, hFGF21 and FAP was exogenously added to the differentiated 3T3/L1 adipocytes in the presence or absence of BR103354. Treatment with hFGF21 increased p-ERK, Glut1 and adiponectin levels, which were reduced in the presence of FAP. BR103354 restored p-ERK levels to those of normal, in parallel with enhanced Glut1 levels (Fig. 2), suggesting that BR103354 was effective as a FAP inhibitor at the cellular level.

### Pharmacokinetic analysis of BR103354

Pharmacokinetic properties of BR103354 were determined in vitro and in vivo for the evaluation of its in vivo efficacy. In the in vitro ADMET study, BR103354 showed satisfactory profiles, including cardiac safety as determined by hERG assay (IC_50_ > 787.5 µM), good plasma and microsomal stability, and little inhibition of 5 CYP isoforms (IC_50_ > 50 µM) (Table 2). Based on in vivo pharmacokinetic measurements, BR103354 appeared to be rapidly absorbed with T_max_, ranging from 0.5 h to 1.1 h in mice and rats, and 2.3 h in monkeys. The half-life (T_1/2_) was 1.2–4.0 h in mice, 3.5 h in rats, and 1.3–2.8 h in monkeys. Oral bioavailability was found to be 48.4% in mice (Table 3).

**Table 2:** In vitro ADMET profiles of BR103354.

Parameters	Results
hERG (IC_50,_ µM)	> 787.5
Microsomal stability (% remaining at 60 min) (H/Mk/D/R/M)	95/81/ > 100/87/98
Plasma stability (% remaining at 60 min) (H/R/M)	> 100/79/94
Plasma protein binding (% bound) (H/R/M)	67/60/78
CYPs inhibition (IC_50_, µM) (H/Mk/D/R/M)	> 50/ > 50/ > 50/ > 50/ > 50
Thermodynamic solubility (pH 1.0/3.0/7.0, µg/mL)	197/165/141
Kinetic solubility (pH 1.0/3.5/7.4, µg/mL)	79/81/78

*H* human,* Mk* monkey,* D* dog,* R* rat,* M* mouse,* CYPs* 1A2, 2C9, 2C19, 2D6, 3A4.

**Table 3:** In vivo PK profiles of BR103354.

Species	Parameters	Results
Mouse	T_max_ (h, 1/5/10/100/200 mg/kg)	0.5/0.5/0.5/1/1
	C_max_ (µg/mL, 1/5/10/100/200 mg/kg)	2.3/7.2/19.2/66.0/122.5
	T_1/2_ (h, 1/5/10/100/200 mg/kg)	1.2/1.1/1.2/4.0/3.8
	AUC_(0–6 h)_ (µg·h/mL, 1/5/10/100/200 mg/kg)	3.7/10.8/41.1/17.0/29.2
	Bioavailability (%)	48.4
Rat	T_max_ (h, 10 mg/kg)	1.1
	C_max_ (µg/mL, 10 mg/kg)	4.4
	T_1/2_ (h, 10 mg/kg)	3.5
	AUC_(0–6 h)_ (µg·h/mL, 10 mg/kg)	19.7
Monkey	T_max_ (h, 10/30 mg/kg)	2.3/2.3
	C_max_ (µg/mL, 10/30 mg/kg)	0.42/2.0
	T_1/2_ (h, 10/30 mg/kg)	1.3/2.8
	AUC_(0–6 h)_ (µg·h/mL, 10/30 mg/kg)	1.2/6.4

### Acute in vivo effects of BR103354 in ob/ob mice

**Figure 3:**
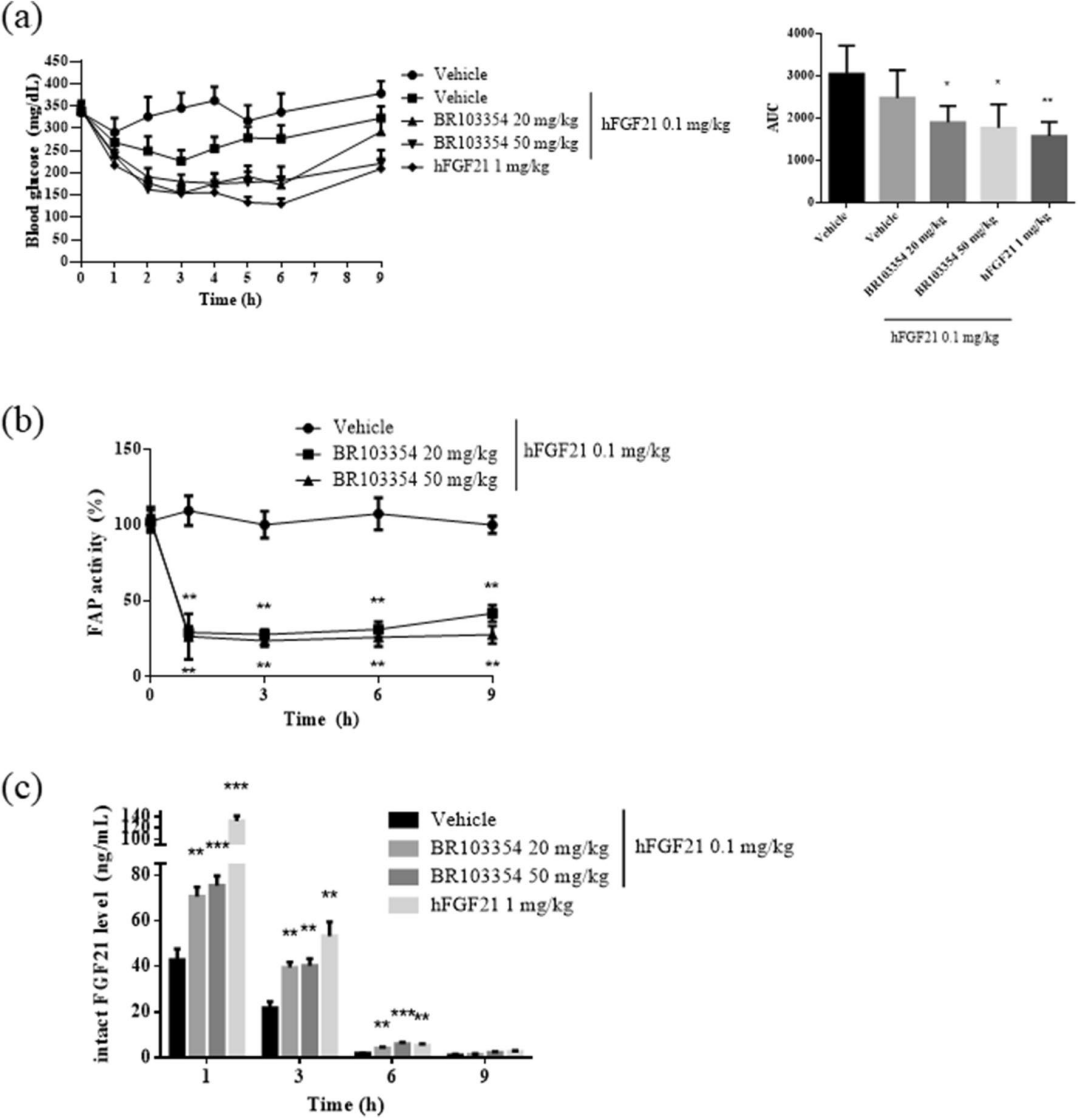
BR103354 acutely reduces blood glucose levels via FAP inhibition in ob/ob mice. 8-week-old ob/ob mice (n = 5–9/group) were treated with BR103354 or vehicle before 15 min *i.p.* treatment of hFGF21. Blood was collected at the indicated time points. (**a**) Nonfasting blood glucose levels were measured at the indicated time points. AUC values are shown for comparison between treatment groups. (**b**) Plasma FAP activity was measured by fluorometric assay using AMC substrate. (**c**) Plasma intact hFGF21 levels were measured using ELISA kit at 1, 3, 6, and 9 h. All data are the means ± S.D. *P < 0.05, **P < 0.01 and *** P < 0.001 vs. vehicle group.

To evaluate any acute metabolic consequences of BR103354, ob/ob mice were orally administered with 20 or 50 mg/kg of BR103354 in combination with 0.1 mg/kg of hFGF21. As a control, 0.1 mg/kg or 1 mg/kg of hFGF21 alone was intraperitoneally administered to the mice. Intraperitoneal injection of 0.1 mg/kg of hFGF21 alone reduced blood glucose levels, starting from 340 mg/dL to a maximum average of 230 mg/dL at 3 h after administration, while AUC decreased by about 20%. BR103354 co-administration further reduced blood glucose levels to an average of 178 mg/dL in the 20 mg/kg-treated group and 154 mg/dL in the 50 mg/kg-treated group at 3 h after administration, while AUC decreased by about 40–45% (Fig. 3a), which was comparable to the effect of 1 mg/kg of hFGF21 alone. BR103354 inhibited FAP activity by more than 70% in 1 h, which was maintained up to 9 h (Fig. 3b), paralleled with increased levels of intact FGF21 in plasma until 3 h (Fig. 3c). However, the levels of intact FGF21 were lower than 10 ng/mL after 6 h of administration. These results suggested that single oral administration of BR103354 prevented the cleavage of hFGF21 via inhibition of endogenous mouse FAP activity in vivo, thereby resulting in reduced plasma glucose levels in ob/ob mice.

### Chronic anti-diabetic effects of BR103354 using ob/ob mice

**Figure 4:**
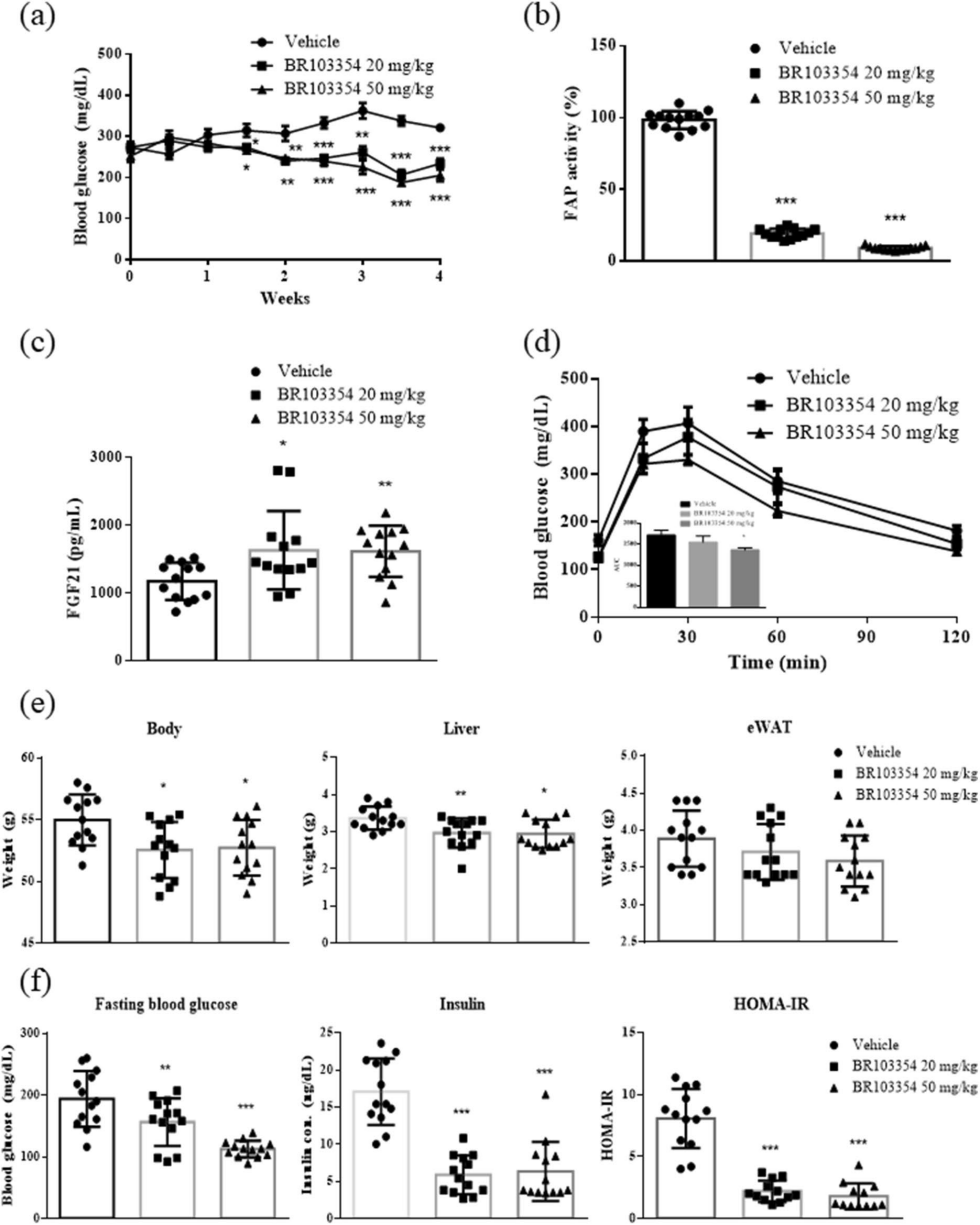

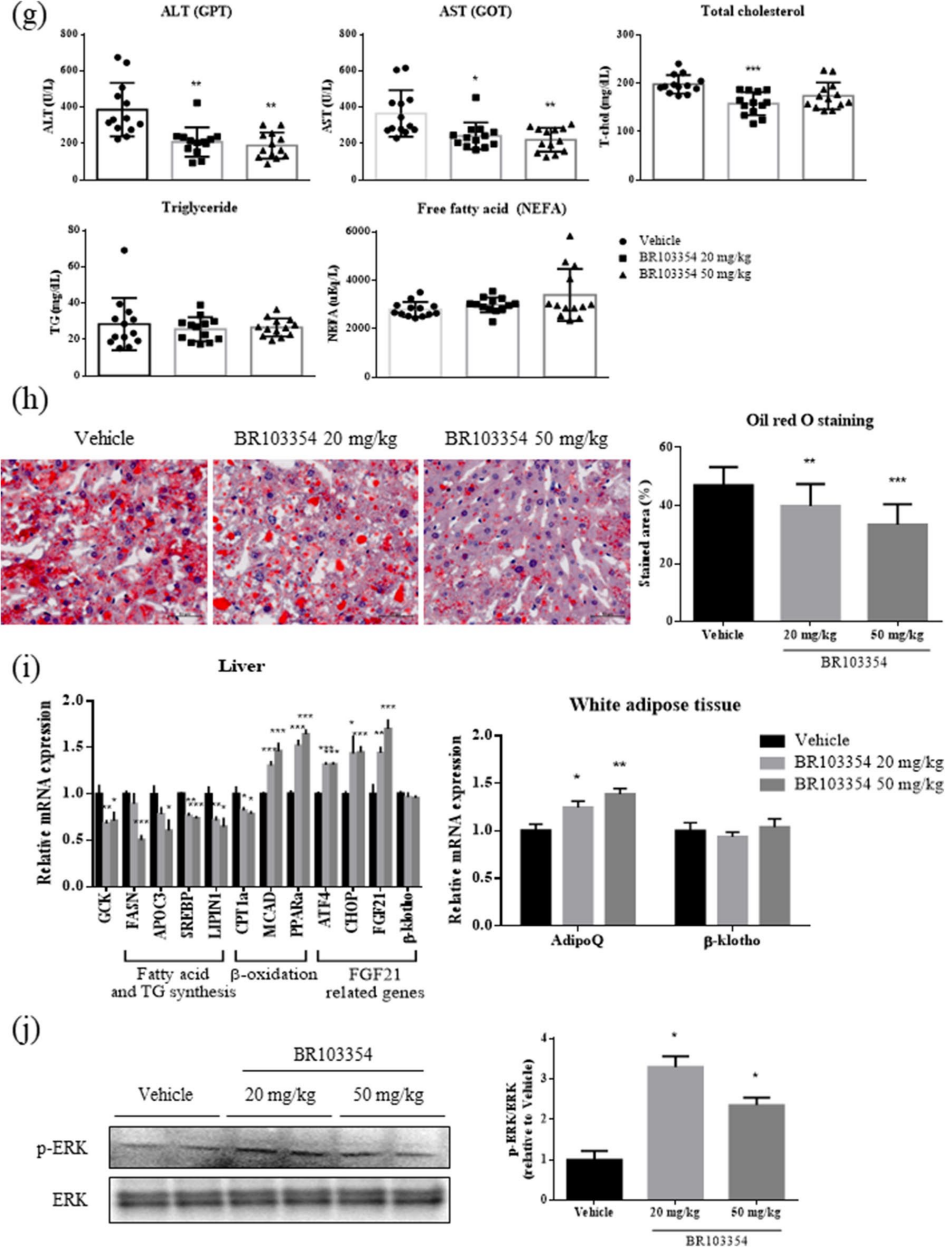
Chronic anti-diabetic effect of BR103354 in ob/ob mice. Male ob/ob mice were treated with vehicle or BR103354 (20 and 50 mg/kg) for 4 weeks (n = 13/group). (**a**) Nonfasting blood glucose levels in ob/ob mice. (**b**) Plasma FAP activity was measured by fluorometric assay using AMC substrate at week 4. (**c**) Serum mouse FGF21 levels at week 4 (**d**) OGTT and AUC in ob/ob mice. (**e**) Fasting body weight, liver weight and epididymal fat weight from ob/ob mice. (**f**) Fasting blood glucose, insulin concentrations and HOMA-IR in ob/ob mice. (**g**) The plasma levels of ALT (GPT), AST (GOT), TG, total cholesterol and NEFA in ob/ob mice. (**h**) Oil red O staining of the liver from ob/ob mice (Scale bar = 50 µm). (**i**) Relative expression of genes related to intrahepatic lipogenic gene (GCK), fatty acid and TG synthesis genes (FASN, SREBP and APOC3, LIPIN1), β-oxidation genes (CPT1α, MCAD and PPARα) and FGF21 related genes (ATF4, CHOP, FGF21 and β-klotho) in liver and FGF21 related genes (AdipoQ and β-klotho) in white adipose tissue of ob/ob mice. (**j**) Immunoblots of p-ERK/ERK of epididymal white adipose tissue lysates from ob/ob mice. The graph shows densitometric quantification of western blot bands. All data are the means ± S.D. *P < 0.05, **P < 0.01 and ***P < 0.001 vs. vehicle group.

Recently, FAP-deficient mice have been reported to exhibit improved insulin resistance, glucose tolerance, and liver steatosis^38^. Additionally, administration of a known FAP inhibitor (Talabostat, TB) to diet-induced obese mice improved insulin resistance and increased endogenous intact FGF21 level^36^. Therefore, we examined the chronic effects of BR103354 in vivo using ob/ob mice without exogenous hFGF21 administration. From 2 weeks after BR103354 administration, blood glucose was significantly decreased in the compound-treated groups compared to the vehicle group (*P* < 0.01), further being reduced at week 3 (*P* < 0.001, Fig. 4a). After 4 weeks of treatment, the FAP activity was found to be inhibited at least to 80% of that in the vehicle group (Fig. 4b). In accordance, total form of serum FGF21 was also increased by approximately 1.4 fold in BR103354-treated groups (Fig. 4c). BR103354 (50 mg/kg) improved glucose tolerance significantly, as determined by the oral glucose tolerance test (Fig. 4d). There was no change in body weight between treatments (data not shown), but fasting body weights, liver weights, fasting blood glucose levels, and insulin levels were significantly reduced after 4 weeks of treatment (Fig. 4e). As determined by HOMA-IR, insulin resistance appears to be ameliorated after BR103354 treatment (Fig. 4f) with increased adiponectin level in white adipose tissues (Fig. 4i). Serum alanine aminotransferase (ALT), aspartate aminotransferase (AST), and total cholesterol were significantly reduced by BR103354 compared to those of the vehicle group, but serum TG and non-esterified free fatty acid (NEFA) levels were unaltered (Fig. 4g).

In association with reduced liver weight by BR103354, liver TG content was decreased, as determined by Oil red O staining (Fig. 4h), along with reduced mRNA expression of genes involved in lipogenesis, steatosis and lipid metabolism, but with increased fatty acid oxidation-related genes such as MCAD and PPARα (Fig. 4i). In addition, increased FGF21, ATF4 and CHOP mRNA expression was observed (Fig. 4i), suggesting that BR103354 induced ATF4-mediated FGF21 transcription. In correlation with in vivo FAP inhibitory effects of BR103354, ERK phosphorylation was increased in the adipose tissues of BR103354-treated groups compared to that of the vehicle group (Fig. 4j), consistent with the results in 3T3/L1 adipocytes, suggesting that the beneficial effects of BR103354 in vivo may be attributable to its inhibitory action on FAP.

### Effects of BR103354 in non-human primates

Mouse FGF21 is resistant to FAP-mediated cleavage at the C-terminus, but in the case of monkeys, FGF21 cleavage at the C-terminus of FGF21 occurs similar to that observed in humans^29^. Therefore, we confirmed the effects of BR103354 on the intact FGF21 levels in a cynomolgus monkey model.

**Figure 5:**
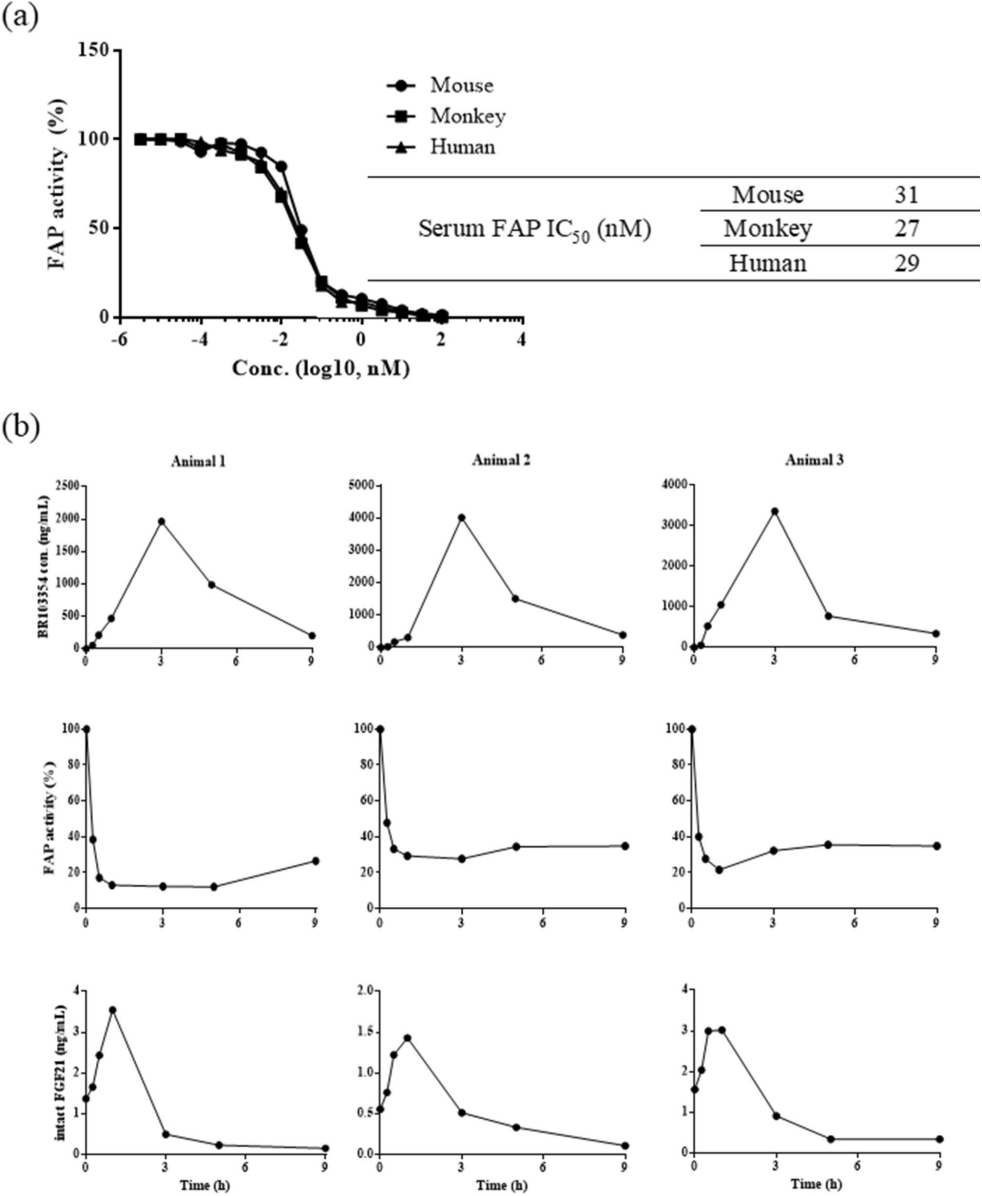
The effect of BR103354 on FAP inhibition and endogenous intact FGF21 levels in non-human primates. (**a**) IC_50_ values of BR103354 using serum FAP of mice, monkeys, and humans were determined. (**b**) Normal cynomolgus monkeys were treated with BR103354 by intranasal injection, and blood was collected at the indicated time points. The plasma concentration of BR103354 was quantified by LC–MS/MS, and plasma FAP activity was measured by fluorometric assay using AMC substrate. Intact FGF21 levels in plasma were measured by ELISA.

The IC_50_s of BR103354 on serum FAP from mice, monkeys, and humans were estimated, and revealed that serum FAP IC_50_ values were similar in all the three species, being approximately 27–31 nM (Fig. 5a). Normal cynomolgus monkeys (n = 3) were treated with BR103354 (50 mg/kg) by intranasal injection, and the compound concentrations in blood were determined by LC–MS/MS at the indicated time points. The concentrations of BR103354 peaked at 3 h after injection in all animals, but the inhibition of FAP activity, as well as the concentrations of intact FGF21, was maximally achieved at 1 h after administration, thus showing an inhibition of 65–83% and 1.5–3.5-fold increase in each animal, respectively (Fig. 5b).

### Effects of BR103354 in a NASH mouse model

**Figure 6:**
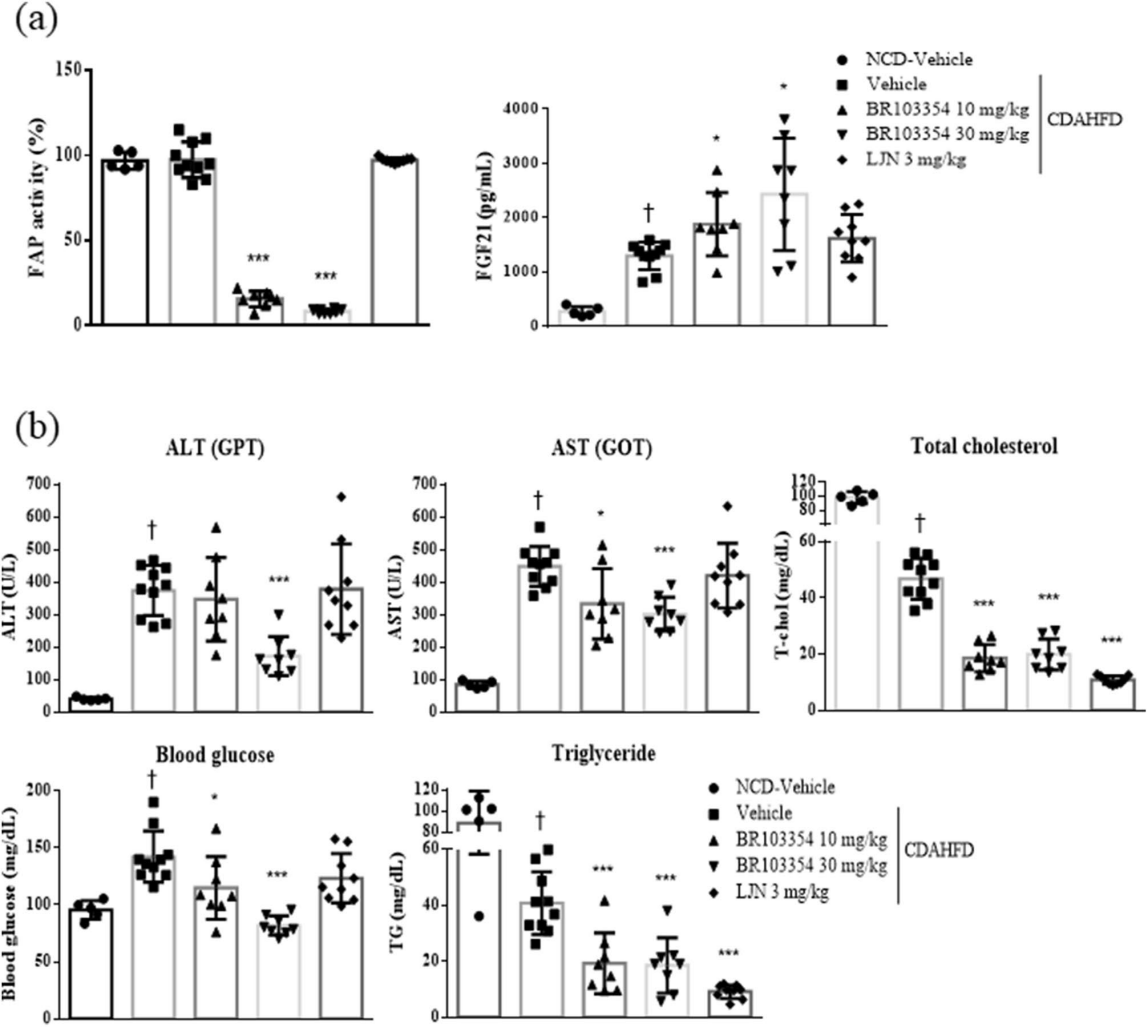

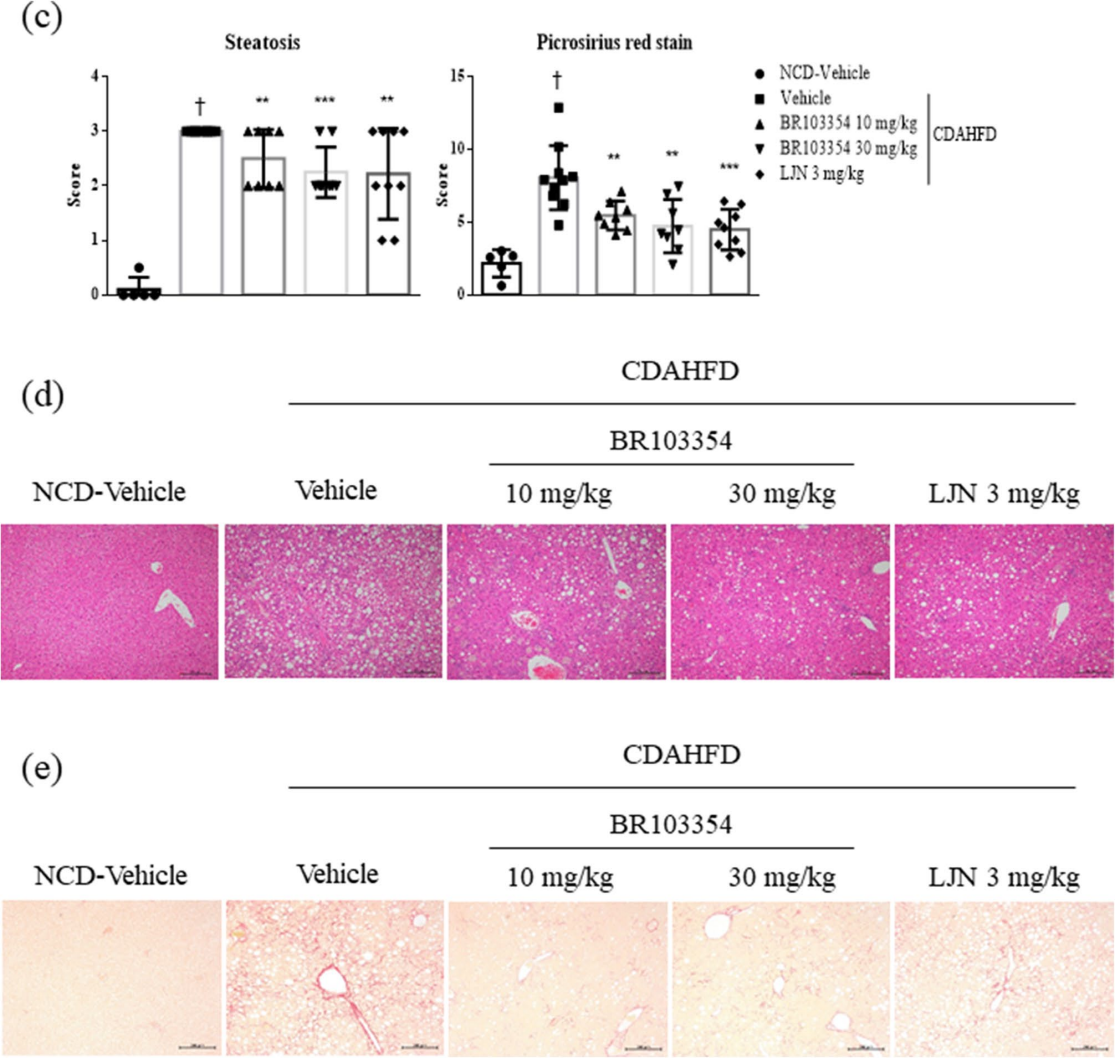
Effects of orally administered BR103354 in CDAHFD mice. C57BL/6J mice were fed normal chow diet (n = 5) or choline-deficient, L-amino acid-defined, high-fat diet (CDAHFD) for 4 weeks to induce NASH (n = 8–10/group). The vehicle, BR103354 (10 and 30 mg/kg) and LJN (positive control, 3 mg/kg) were orally administered for 10 weeks. (**a**) Relative FAP activity was assessed by FRET-quench and serum FGF21 level at week 14. (**b**) The plasma levels of ALT (GPT), AST (GOT), total cholesterol, glucose and TG in NCD and CDAHFD mice were measured at week 14. (**c**) Steatosis scores ranged from 0 to 3 (normal = 0, minimal = 1, moderate = 2, marked = 3) were determined. Hepatic steatosis and fibrosis (Picrosirius red stain) were analyzed in a blinded manner or by digital image analyzer. (**d**–**e**) Histological evaluations of liver tissue were performed in each group. Paraffin-embedded liver sections were stained with H&E (**d**) and Sirius red (**e**). Representative photographs of each group were presented. Images were obtained at 100x (Scale bar = 50 µm). All data are the means ± S.D. *P < 0.05, **P < 0.01 and ***P < 0.001 vs. CDAHFD-vehicle group and †*P* < 0.001 vs. NCD-vehicle group.

Since BR103354 improved liver steatosis in ob/ob mice after 4 weeks of treatment (as shown in Fig. 4), the beneficial effects of BR103354 on liver steatosis were confirmed using a CDAHFD NASH mouse model. After 10 weeks treatment with BR103354, FAP activity was found to be inhibited in a dose-dependent manner, as compared to vehicle-treated group, in association with increased serum FGF21 levels by about 1.5–1.9 folds in BR103354 treatment groups (Fig. 6a). Additionally, blood ALT and AST levels, total cholesterol, TG, and glucose levels were all reduced in BR103354-treated groups, as compared to those in the vehicle group (Fig. 6b). Steatosis scores as well as Picrosirius red staining also showed significant reduction in liver fibrosis in the BR103354-treated groups (Fig. 6c,e), as compared to that exhibited by hematoxylin and eosin (H&E) staining (Fig. 6d). LJN (Tropifexor, FXR agonist, 3 mg/kg) was used as a positive control. All parameters showed improvements in the study.

## Discussion

FGF21 is an endocrine hormone that plays an important role in energy homeostasis, insulin resistance, dyslipidemia, as well as fatty liver disease, and elevation of FGF21 action is an attractive, new therapeutic strategy for the treatment of metabolic diseases including diabetes^4–7^. Despite extensive efforts by global pharmaceutical companies to develop FGF21 analogues, several clinical trials failed to produce positive outcomes for the treatment of type 2 diabetes and its related metabolic disorders^19,28,39^.

FAP, an enzyme in the DPP protease family, was identified as the cleavage enzyme responsible for FGF21 inactivation, leading to disruption of its binding to β-klotho, an obligatory FGF21 co-receptor. Correspondingly, FAP knockout mice fed with a high-fat diet improved insulin resistance and glucose tolerance in the liver and adipose tissue, similar to those observed in the FGF21 transgenic mice^38^. Based on these findings, several FAP inhibitors were reported and showed increasing levels of intact FGF21, accompanied by improved insulin resistance and glucose tolerance upon in vivo administration^36^, thus providing proof of concept of FAP inhibition as a new therapeutic approach.

To discover novel and potent FAP inhibitors in our study, we screened > 800 compounds from a chemical library and identified BR103354 as a potential candidate for the treatment of diabetes and metabolic diseases. The in vitro inhibitory effects of BR103354 on FAP were confirmed in various assays: shift in molecular weight by SDS-PAGE after incubation with hFGF21 and FAP, and fluorescence determination with AMC-coupled substrate using hFGF21 or serum samples from different species. All measurements revealed comparable potency levels for BR103354, specifically IC_50_ values of approximately 10–30 nM.

To determine in vivo efficacy of BR103354, several animal models were employed in the current study. First, the acute effects of BR103354 were examined in ob/ob mice by single co-administration of the compound with exogenous hFGF21, since endogenous mouse FGF21 is resistant to FAP cleavage. As expected, intraperitoneal injection of hFGF21 lowered blood glucose levels, the effects of which were further enhanced in the presence of BR103354 in a dose-dependent manner. Correspondingly, FAP inhibition as well as increased intact FGF21 levels were observed, suggesting that BR103354 indeed inhibited FAP-mediated hFGF21 cleavage, thus resulting in the reduction of blood glucose in ob/ob mice. The inhibitory effects of BR103354 on FAP activity were maintained for 9 h after single administration, but increased intact FGF21 levels disappeared at about 6 h. This discrepancy might be due to the slow dissociation of BR103354 from FAP and/or the inability to slow down renal clearance of hFGF21.

Chronic administration of BR103354 to ob/ob mice ameliorated various metabolic parameters such as blood glucose and insulin levels, body and liver weights, ALT and AST, and total cholesterol levels. Since we have not administered hFGF21 exogenously and mouse FGF21 is resistant to FAP-induced cleavage, it is unclear whether the beneficial effects of BR103354 in ob/ob mice observed in our study can be attributed to BR103354-induced increases of intact FGF21. Similarly, animals administered with Talabostat alone, a non-selective FAP inhibitor, displayed decreased body weight, food consumption and adiposity, but with improved insulin sensitivity, in parallel with increased intact FGF21 levels^36^. Interestingly, increased mRNA expression of FGF21 was detected in livers of the BR103354-treated groups, in parallel with increased CHOP expression, both of which were regulated by ATF4 transcription factor, suggesting that BR103354 induces transcription factor ATF4, leading to increased hepatic mRNA expression of FGF21. Although the precise mechanisms involved in the beneficial effects of BR103354 are not clear at present, it may be possible that FAP inhibition in mice may increase the intact form of FGF21 via blockade of the N-terminus cleavage, and this increase may interfere with receptor-mediated or renal clearance, producing metabolically beneficial effects. Alternatively, increased hepatic FGF21 may contribute to the beneficial effects of BR103354, in agreement with the finding of Chowdhury et al.^38^. Since FGF21 production by brown adipose tissue may be increased under our experimental condition, possible involvement of FGF21 as a batokine in the action of BR103354 cannot be excluded at present. Further studies are needed for these issues.

In order to further confirm the inhibitory effects of BR103354 on FAP in vivo, BR103354 was given to cynomolgus monkey, a non-human primate species having a similar profile for FAP-induced FGF cleavage, and the levels of FAP activity and intact FGF21 were determined. Maximum effects on FAP activity and intact FGF21 levels were achieved at 2 h after BR103354 administration, whereas plasma concentrations of BR103354 were highest at 3 h after administration. These results may indicate that even submaximal concentrations of BR103354 in plasma were sufficiently high enough to inhibit FAP activity to the maximum extent. It would be helpful to examine the effects of BR103354 in a preclinical study using a disease monkey model to determine whether the compound is capable of improving metabolic profiles.

Increased FAP expression in activated hepatic stellate cells and in plasma of patients with cirrhosis were reported, thus indicating that FAP could negatively regulate the hepatoprotective activity of FGF21^26^. Under these conditions, FAP inhibition may enhance the protective activity of endogenous FGF21 in liver via regulation of lipid handling and ER stress. Therefore, we induced mild NASH in mice by feeding a CDAHF diet for 4 weeks to examine the effect of BR103354 in this NASH model. In association with FAP inhibition, various parameters of liver function were improved by BR103354 administration, suggesting that BR103354 produces hepatoprotective effects against NASH. Increased serum FGF21 was also detected, and further precise mechanism of liver protective effects by BR103354 awaits elucidation. Additionally, the CDAHFD mouse model is often an inappropriate substitute for human fibrosis, since the characteristics of a choline-deficient diet are known to induce inflammation and fibrosis through liver damage, which is unrelated to the induction of metabolic syndrome and insulin resistance^40^. Moreover, serum total cholesterol and TG levels were significantly lower in CDAHFD mouse model as compared with control mice^41^. On the other hand, mice fed with a western diet have a NASH formation similar to that in humans, so that further evaluation of the liver protective effect of BR103354 would require a more precise mimicking of human NASH formation.

Pharmacokinetic properties of BR103354 were determined, thus showing the proper characteristics for a promising preclinical candidate (F = 48.4%, T_max_ 1 h, T_1/2_ 1.2 h) with cardiac safety and weak CYP inhibitory effects. There are some limitations on the current study to be answered. Since rodent FGF21 is resistant to FAP-mediated cleavage, the precise mechanisms involved in the beneficial effects of BR103354 should be further clarified. Additionally, the potential role of FGF21 produced by brown adipose tissues in BR103354 action needs to be investigated. Taken together, the present study demonstrates that BR103354 acts as a selective and potent FAP inhibitor and improves insulin resistance, glucose tolerance, and hepatic fibrosis in various animal models, possibly via FAP inhibition. Further studies will help to reveal the potential therapeutic usefulness of BR103354 in a range of metabolic disorders.

## Methods

### Recombinant proteins

Recombinant human PREP (hPREP) (catalog no. 4308-SE), hDPP2/7 (catalog no. 3438-SE), hDPP9 (catalog no. 5419-SE), hFAP (catalog no. 3715-SE), and hDPP4 (catalog no. 9168-SE) proteins were from R&D Systems (Minneapolis, MN). Human FGF21 (catalog no. CRF159C) was from Cell Science (Newburyport, MA).

### Determination of IC_50_ value of BR103354

FAP enzyme activities were assayed in 20 mM Tris/HCl, 0.1 M NaCl, 1 mM EDTA (pH 8.0) in the presence of 40 μM Suc-Gly-Pro-Leu-Gly-Pro-AMC (#I-1350.0100, BACHEM, Bubendorf, Switzerland) and hFAP in a total volume of 100 µL. The reaction mixture was incubated in the presence of various concentrations of BR103354 for 60 min at 37 °C, and the fluorescence of AMC released by FAP-induced cleavage was then determined at an emission wavelength of 450 nm with FlexStation 3 (Molecular Devices). The inhibitor concentrations ranged from 100 μM to 0.03 nM, and its IC_50_ values were computed with commercially available curve-fitting programs such as the SigmaPlot. Other protease activities were determined in a similar manner.

### Quantitative real-time RT-PCR

RNA was prepared from liver tissue using the TRIzol Reagent (Invitrogen, Carlsbad, CA). cDNA was synthesized using Superscript II (Invitrogen) and oligo(dT) 12–18 primer. Gene expression was examined by real-time RT-PCR analysis using specific primers as follows; mouse GCK (sense: 5′-CTCTGAGTGCATCTCTGACTTC-3′ and antisense: 5′-TGTCTATGTCTTCGTGCCTTAC-3′), mouse FASN (sense: 5′-AGACCCGAACTCCAAGTTATTC-3′ and antisense: 5′-GCAGCTCCTTGTATACTTCTCC-3′), mouse APOC3 (sense: 5′-AGGCTACTGGAGCAAGTTTAC-3′ and antisense: 5′-CACAGAAGTCTCACGACTCAAT-3′), mouse SREBP (sense: 5′-TTTCCGGGGAACTTTTCCTT-3′ and antisense: 5′-CTTGGTTGTTGATGAGCTGG-3′), mouse LPIN1 (sense: 5′-GGAGCTGCGAGAATGGAAAG-3′ and antisense: 5′-TTCTGCTTCCATCCATCGGT-3′), mouse CPT1α (sense: 5′-TCGAAACCCAGTGCCTTAAC-3′ and antisense: 5′-AAGCAGCACCCTCACATATC-3′), mouse MCAD (sense: 5′-TGCAGATTTTCGGAGGCTAT-3′ and antisense: 5′- GCTTAGTTACACGAGGGTGA-3′), mouse PPARα (sense: 5′- CGAACATTGGTGTTCGCAG-3′ and antisense: 5′-CTTCAACTTGGCTCTCCTCT-3′), mouse ATF4 (sense: 5′-TCTCTAACGCCACAGTTACC-3′ and antisense: 5′- CACAACTTAAACCGGCAGAC-3′), mouse CHOP (sense: 5′-AGAGAGTGTTCCAGAAGGAAG-3′ and antisense: 5′- GCAGGGTCAAGAGTAGTGAA-3′), mouse FGF21 (sense: 5′-GGAGGATGGAACAGTGGTAGGC-3′ and antisense: 5′-AGGCTTTGACACCCAGGATTTG-3′), mouse β-klotho (sense: 5′-TCCGGGGAATGAATGGATTT-3′ and antisense: 5′- GTTTACCGGACTCACGTACT-3′), mouse adipoQ (sense: 5′-CATGCCGAAGATGACGTTAC-3′ and antisense: 5′- TCTCACCCTTAGGACCAAGA-3′) and mouse GAPDH (sense: 5′-AGGTCGGTGTGAACGGATTTG-3′ and antisense: 5′-TGTAGACCATGTAGTTGAGGTCA-3′). GAPDH was used as an internal control. These experiments were assessed by Woojung Bio Inc. (Suwon, Gyeonggi-do, Korea).

### Immunoblot analysis

Western blots were performed as described^42^. For protein preparation, cells or tissues were solubilized in a lysis buffer. Protein concentration was determined using a Bio-Rad Protein Assay kit (Bio-Rad, Richmond, CA). Then, 20 μg of the protein was electrophoresed by SDS-PAGE and electrotransferred to polyvinylidene fluoride membranes (Millipore, Bedford, MA). After blocking with 5% skimmed milk in Tris-buffered saline containing Tween 20 (0.1%) for 1 h, the membrane was incubated with polyclonal antibodies against phospho-ERK (1:1,000; Cell Signaling Tech., Danvers, MA, 4376S), ERK (1:1,000; Cell Signaling Tech., 4695S), Glut1 (1:1,000; AB chem, Cambridge, UK, ab652), adiponectin (1:1000; Cell Signaling Tech., 2789) or β-actin (1:1,000; Cell Signaling Tech., 4967S) at 4 °C with gentle shaking overnight. Antibodies were detected by horseradish peroxidase-linked secondary antibody (1:10,000; Cell Signaling Tech., 7074S), using the enhanced chemiluminescence Western Blotting Detection System (Molecular Imager Gel Doc XR System, BIO-RAD). The intensities of the protein bands were performed with ImageJ 1.52v software. Images of original western blotting are shown in Supplementary Figs. 1–3.

### Compounds and dosing

BR103354, a FAP-specific inhibitor, (S)-N-(2-(2-cyano-4,4-difluoropyrrolidin-1-yl)-2-oxoethyl)-2-(4-cyanobenzyl)thiazole-4-carboxamide, was synthesized by Boryung Pharmaceutical Co. Ltd (Seoul, Korea). The chemical structure of BR103354 is shown in Fig. 1a. BR103354 was formulated in 5% DMSO/45% PEG/50% DW and administered at 10, 20, 30 or 50 mg/kg via oral gavage intubation. Negative control animals received vehicle only.

### Pharmacokinetic study

#### In vitro ADME

All in vitro ADME assays were conducted at the Daegu-Gyeongbuk Medical Innovation Foundation (DGMIF, Dong-gu, Daegu, Korea).

#### hERG binding assay based on fluorescence polarization

Predictor hERG Assay test kits were obtained from Invitrogen (Carlsbad, CA). The binding assay was carried out according to the manufacturer’s instructions. Briefly, aliquots (5 μL) of each concentration of BR103354 were pipetted into the appropriate wells of a 384-well microplate, and 10 μL of (2x) the membrane preparation was then dispensed. Working Tracer solution (5 μL) was then added and the plate was allowed to incubate at room temperature for at least 1 h, and then fluorescence polarization was measured using the Synergy 4 Hybrid Microplate Reader from BioTek Instruments (Winooski, VT) with 530/25 excitation and 590/35 emission filters along with a 570 nm cut off dichroic mirror. Polarization values were calculated automatically using Gen Data Analysis Software (BioTek Instruments). The above protocol is described in detail in the Invitrogen’s application note.

#### Plasma and microsomal stability

Plasma or liver microsomes (0.5 mg/mL), 0.1 M phosphate buffer solution (pH 7.4), and BR103354 at a concentration of 1 µM were combined, and the mixture was pre-incubated at 37℃ for 5 min and then further incubated with NADPH regeneration system solution at 37℃ for 60 min. Aliquots were then transferred in duplicate to: (i) a final concentration plate that was immediately quenched with 23.6 ng/mL internal standard (IS, chlorpropamide) in acetonitrile (ACN) or (ii) tubes, which were centrifuged for 5 min at 4℃ (14,000 rpm). The samples were subjected to LC–MS/MS analysis on an AB Sciex 4000 Q Trap hybrid triple quadrupole linear ion-trap MS (AB Sciex, Framingham, MA) coupled to an Agilent 1200 HPLC (Agilent Tech. Santa Clara, CA) with a reverse-phase Kinetex XB-C18 column (2.1 × 100 mm, 2.6 µm; Phenomenex). Mobile phases were comprised of 0.1% formic acid (FA) in water (A) and 0.1 FA in ACN (B). Data analysis was performed using Xcalibur (Ver. 1.6.1).

#### Protein binding

Protein binding was conducted as described^43^. Compound solutions prepared in DMSO were diluted in phosphate buffer (final concentration: 1 µg/mL), and added into human plasma (total volume: 1 mL). Aliquots were then transferred in duplicate to: (i) a final concentration plate that was immediately quenched with 23.6 ng/mL internal standard (IS, chlorpropamide) in ACN, (ii) a degradation control, which was placed in a water bath at 37℃ for 4 h, and (iii) ultracentrifuge tubes, which were centrifuged for 5 min at 4℃ (14,000 rpm). All reactions were stopped by the addition of ACN containing 23.6 ng/mL chlorpropamide. The samples were subjected to LC–MS/MS analysis.

#### CYPs inhibition

Human liver microsome (0.25 mg/mL) was incubated with substrate cocktail (phenacetin 50 µM, diclofenac 10 µM, S-mephenytoin 100 µM, dextromethorphan 5 µM, and midazolam 2.5 µM) and various concentrations of BR103354 in NADPH regeneration system solution at 37℃ for 15 min. Metabolites of the substrate probes were simultaneously analyzed by multiple-reaction monitoring using routine LC-MS/MS.

#### Kinetic solubility

Kinetic solubility was conducted as described^43^. Stock solutions of each compound prepared in DMSO (10 mM) were spiked in duplicate into phosphate buffer at pH 7.4 (final concentration: 200 µM). The samples were agitated for 2 h using a plate shaker (500 rpm, 22℃). Approximately 150 µL of the resulting supernatant was transferred to a 96-well analysis plate and the calibration and test samples were analyzed by HPLC with a diode array detector (Agilent 1200 HPLC) comprising a reverse-phase Kinetex XB-C18 column (2.1 × 100 mm, 2.6 µm; Phenomenex). Mobile phases were composed of 0.1% FA in water (A) and 0.1% FA in ACN (B).

### In vivo ADME

#### In mice

A pharmacokinetic study of BR103354 was performed using C57BL/6J 7-week-old male mice (23–25 g). Mice were administered with BR103354 either *p.o.* (1, 5, 10, 100 or 200 mg/kg) or *i.v.* (2 mg/kg). Blood samples (approximately 0.2 mL) were collected at harvesting of one blood per point from the orbital blood at 0.5, 1, 2, 4, and 6 h (*p.o.*), and 0.02, 0.08, 0.167, 0.33, 0.5, 1, 2, 4, 6, and 8 h (*i.v.*) after administration of BR103354. These samples were then centrifuged at 13,000 rpm for 10 min to obtain plasma. The plasma was divided into 1.5-mL microtubes and stored in a deep freezer at − 80 to − 60 °C until analysis. Samples were analyzed on an API 4500 Qtrap LC‐MS/MS system (Applied Biosystems/MDS Sciex, Toronto, Canada) coupled with Agilent 1200 HPLC (Agilent Technologies, Santa Clara, CA). Plasma concentration–time data were analyzed by non-compartmental method using the nonlinear least-squares regression program WinNonlin (Pharsight, Mountain View, CA).

#### In rats

Six male Sprague–Dawley rats (7-week-old, 240–250 g) were obtained from Orient Bio INC. (Seongnam, Gyeonggi-do, Korea) and maintained at 22℃ on a 12-h light/12-h dark cycle with free access to water. All animals were maintained under such conditions for a minimum of 2 weeks prior to initiation of the studies. After an overnight fasting, rats were orally administered with BR103354 (10 mg/kg). Blood samples (approximately 0.5 mL) were collected from the jugular vein at 0.167, 0.33, 0.67, 1, 2, 4, 6, and 9 h after administration of BR103354. Plasma sample preparations and analysis were the same as described in mice study.

#### In monkeys

Six male cynomolgus monkeys (4.5–5.5 kg) were obtained from Sichuan Primed Shines Bio-tech Co., Ltd (Chengdu, Sichuan, China) and maintained at 18–26 °C, a relative humidity of 40%-70%, a minimum of 8 air changes/h, and a 12-h light/12-h dark cycle. After an overnight fasting, monkeys were administered with BR103354 (10 or 30 mg/kg) intranasally. Blood samples (approximately 1 mL) were collected from the femoral vein at 0.15, 0.5, 1, 3, 5, and 9 h after administration of BR103354. Plasma sample preparations and analysis were the same as described in mice study.

#### Animals and diets

Maintainment of animals was conducted as described^42^. For acute and chronic in vivo studies, 8-week-old male ob/ob mice (Jackson Laboratory) were maintained in a 12-h light/12-h dark cycle and fed with a chow diet. For the NASH mice model, 5-week-old male C57BL/6J mice (Jackson Laboratory) were fed with a CDAHFD consisting of 60% kcal fat and 0.1% methionine. All animals were maintained under such conditions for a minimum of 2 weeks prior to initiation of the pharmacological studies. Before the beginning of the studies, mice were randomized into treatment groups according to body weight and blood glucose levels. All injections and treatments were performed during the light cycle.

### In vivo studies

#### Acute study using ob/ob mice

For the determination of the acute effects of BR103354, ob/ob mice were orally administered with BR103354 (20 or 50 mg/kg) and then treated with 0.1 mg/kg of hFGF21 (*i.p.*) (low dose) 15 min after BR103354 administration or after administration of 1 mg/kg of hFGF21 alone (high dose, positive control) (n = 5–9/group). Tail blood glucose levels were determined using a glucometer (Accu-Chek Active; Roche Diagnostics, Basel, Switzerland) before (0) and 1, 2, 3, 4, 5, 6, and 9 h after BR103354 administration. Blood was promptly collected by orbital bleeding that was allowed to clot, and supernatant was collected after centrifugation at 4 °C at 3000 rpm. Serum intact FGF21 concentrations were measured by intact hFGF21 ELISA (Alpco, Keewaydin Dr, Salem, NH, catalog no. 43-FGFHU-E01)^29^. Serum FAP activity was measured using a six-residue FRET-quench substrate corresponding to the residues flanking the hFGF21 C-terminal cleavage site (i.e. VGP SQG).

#### Chronic study using ob/ob mice

BR103354 (20 or 50 mg/kg) or vehicle was administered orally to 8-week-old male ob/ob mice once daily for 4 weeks (n = 13/group). Serum FAP activity was measured at 4 weeks using a six-residue FRET-quench substrate corresponding to the residues flanking the hFGF21 C-terminal cleavage site. Serum FGF21 concentrations were measured by mouse FGF21 ELISA (R&D Systems, Minneapolis, MN, catalog no. MF2100). During the observation period, mice were monitored for glucose profiles and weighed. OGTT was performed by oral gavage of 1 g/kg glucose after overnight fasting, and then glucose concentrations from the tail vein were determined using a glucometer (Accu-Chek Active; Roche Diagnostics) before (0 min) and 15, 30, 60, and 120 min after glucose treatment. HOMA-IR (Homeostatic Model Assessment of Insulin Resistance) was calculated using the following formula: (fasting insulin × fasting glucose)/22.5, as described previously^44^. The mice were subjected to a 16-h fast before harvesting. Under anesthesia, blood was promptly collected by orbital bleeding and then harvested. Liver tissue was then collected. Serum ALT, AST, TG, total cholesterol, and NEFA levels were assessed by KNOTUS (Incheon, Korea). For light microscopy (Eclipse Meta Morph V 5.0.7; Nikon, West Lafayette, IN) analysis of H&E-stained liver sections, liver tissue was fixed with phosphate-buffered saline containing 4% paraformaldehyde for overnight and then embedded in paraffin. Sections (4 μm) were cut and deparaffinized in xylene, followed by rehydration in a graded series of ethanol. To visualize lipid droplets in the liver, the frozen liver sections were collected and were subjected to Oil red O staining as described previously^45^ (assessed by KNOTUS).

#### In vivo study using cynomolgus monkeys

An in vivo study using cynomolgus monkey was conducted at Sichuan PriMed Shines Bio-tech Co., Ltd (China). Cynomolgus monkeys (n = 3) were treated with BR103354 (50 mg/kg) intranasally, and blood samples were collected pre-and post-dose from the femoral vein at 0.25, 0.5, 1, 3, 5, and 9 h. The plasma concentration of BR103354 was quantified using a qualified LC–MS/MS method (lower limit of quantification, 5 nM). FAP activity and intact FGF21 concentrations were measured as described above.

#### In vivo study using CDAHFD mice

Male C57BL/6J mice (5 weeks of age) were fed with a CDAHFD consisting of 60% kcal fat and 0.1% methionine. BR103354 (10 or 30 mg/kg) and LJN 3 mg/kg (positive control) were administered once daily by oral gavage (n = 8–10/group). For the CDAHFD model, compound treatments were started at the beginning of week 4 on the diet and continued until week 14. FAP activity was measured at 14 weeks using a six-residue FRET-quench substrate corresponding to the residues flanking the hFGF21 C-terminal cleavage site. Serum FGF21 concentrations were measured by mouse FGF21 ELISA (R&D Systems, Minneapolis, MN, catalog no. MF2100). The mice were subjected to a 16-h fast before harvesting. Under anesthesia, blood was promptly collected by orbital bleeding, and the animal was then harvested. Liver tissue was then collected. All other measurements were done similarly as described above. Staining was performed using H&E and Picrosirius red as described previously^46^. Areas of positive Sirius red were quantified by computer-based morphometric analysis.

#### Ethics statement

All animal experiments were carried out in accordance with the animal research guidelines for the Care and Use of Laboratory Animals from US National Instisute of Health (NIH) and were approved by Boryung Pharmaceutical Institutional Animal Care and Use Committee (IACUC).

#### Statistical analysis

All values were expressed as means ± S.D. Two-tailed Student’s *t*-test was used to compare the values between two groups and one-way ANOVA was used for comparisons among groups. A p value of less than 0.05 was considered significant.

## Supplementary information

Supplementary Figures.
