# Supplementary material for: Discovery of a novel fibroblast activation protein (FAP) inhibitor, BR103354, with anti-diabetic and anti-steatotic effects
Source: Sci Rep. 2020 Dec 4;10:21280. doi: 10.1038/s41598-020-77978-z (PMC7718273; doi:10.1038/s41598-020-77978-z)
Supplement: Supplementary file 1 — Supplementary Figures. [file 41598_2020_77978_MOESM1_ESM.pdf]

Discovery of a novel fibroblast activation protein (FAP) inhibitor, BR103354, with anti-diabetic and anti-steatotic effects

Jae Min Cho[1,2], Eun Hee Yang[1], Wenying Quan[1], Eun Hye Nam[1,]

Hyae Gyeong Cheon[2*]

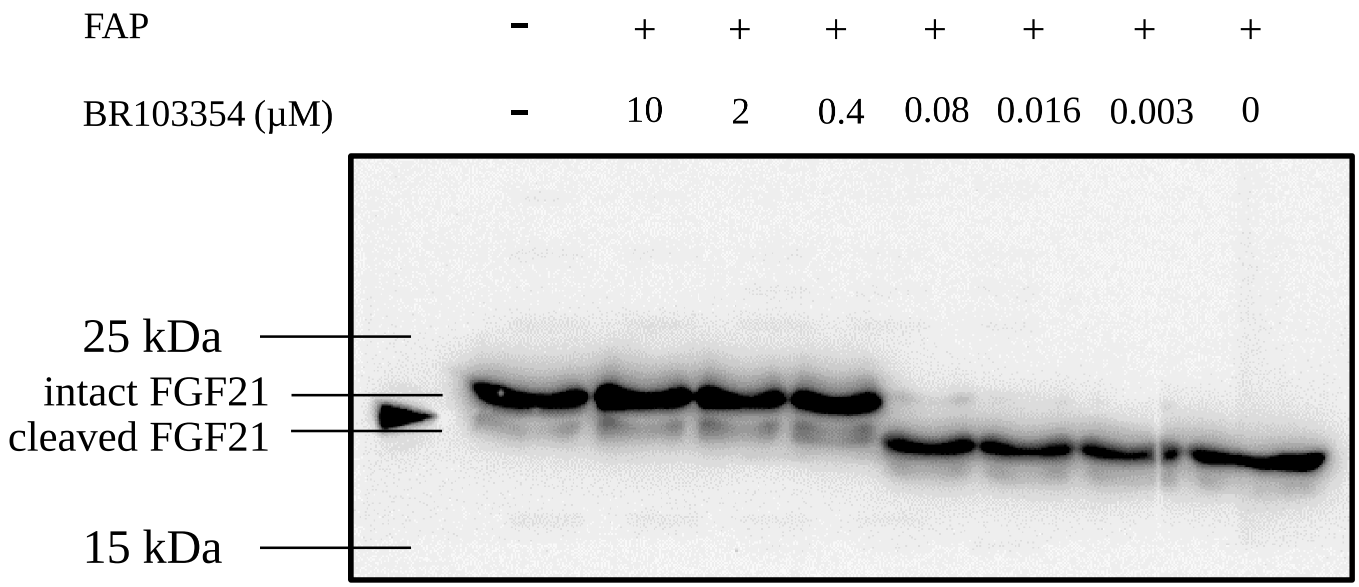

hFGF21 expected size 20.6 kDa

Supplementary Figure 1. Uncropped scans of western blot displayed in Fig. 1c.

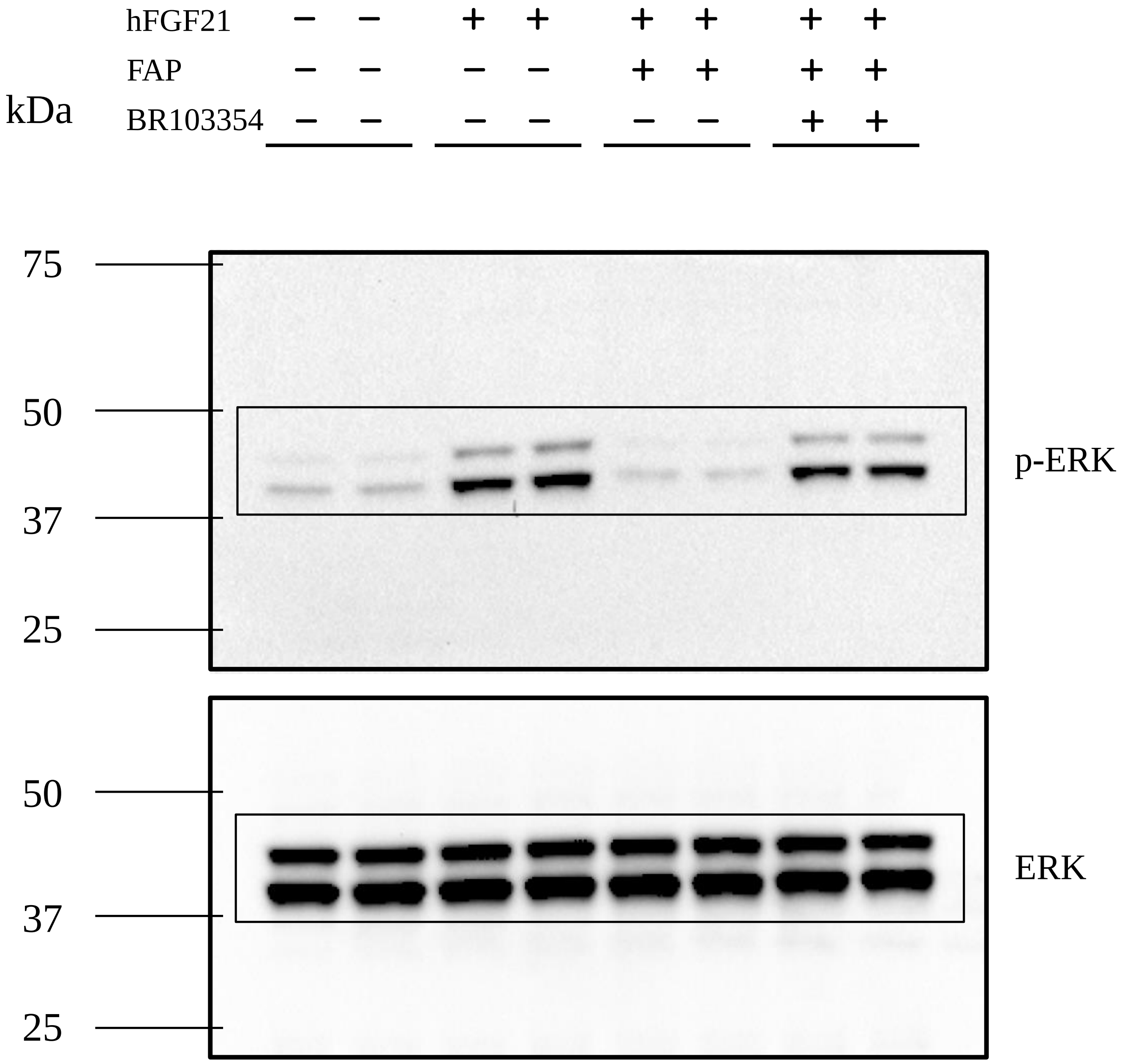

Supplementary Figure 2. Uncropped scans of western blot displayed in Fig. 2.

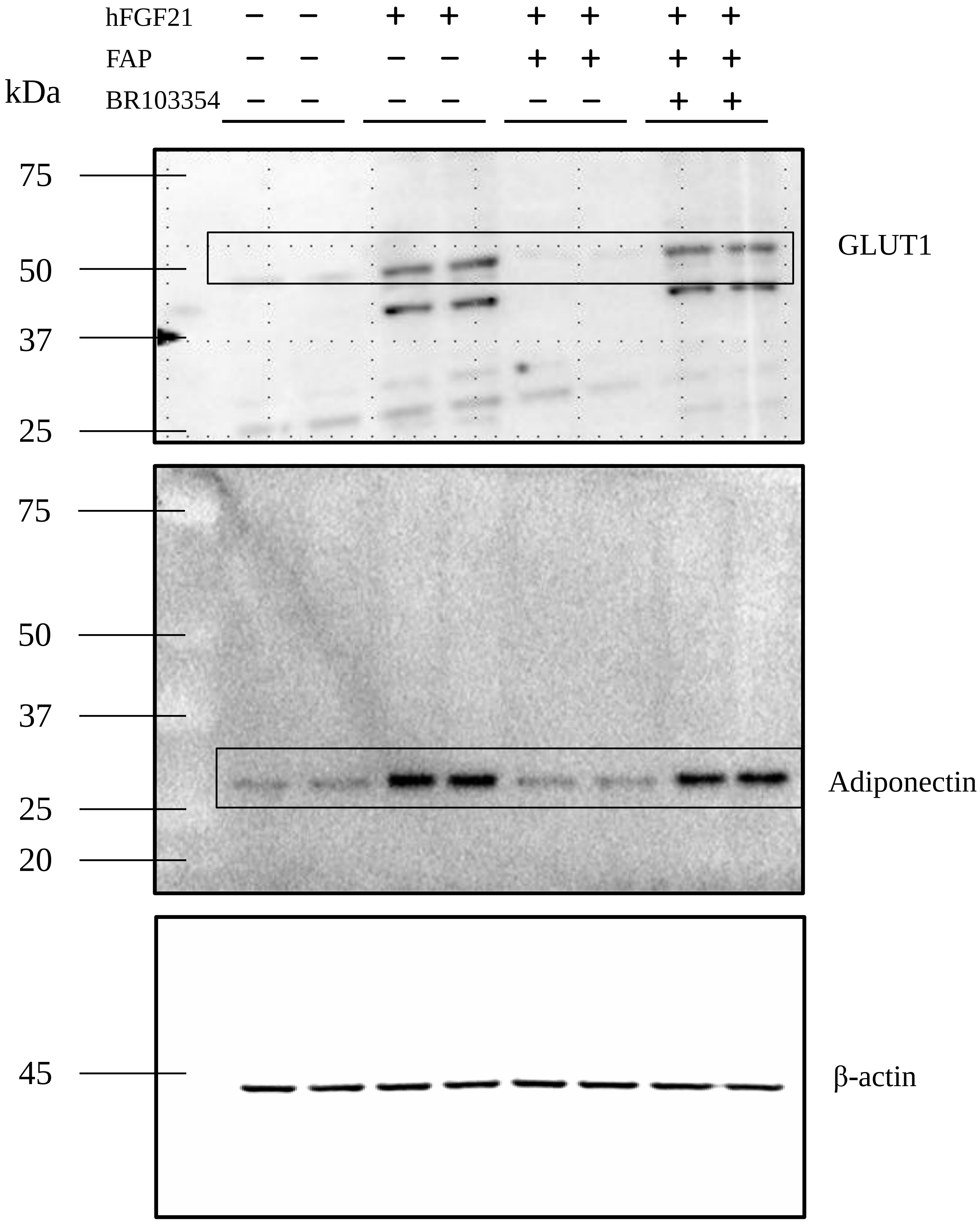

Supplementary Figure 2. Uncropped scans of western blot displayed in Fig. 2.

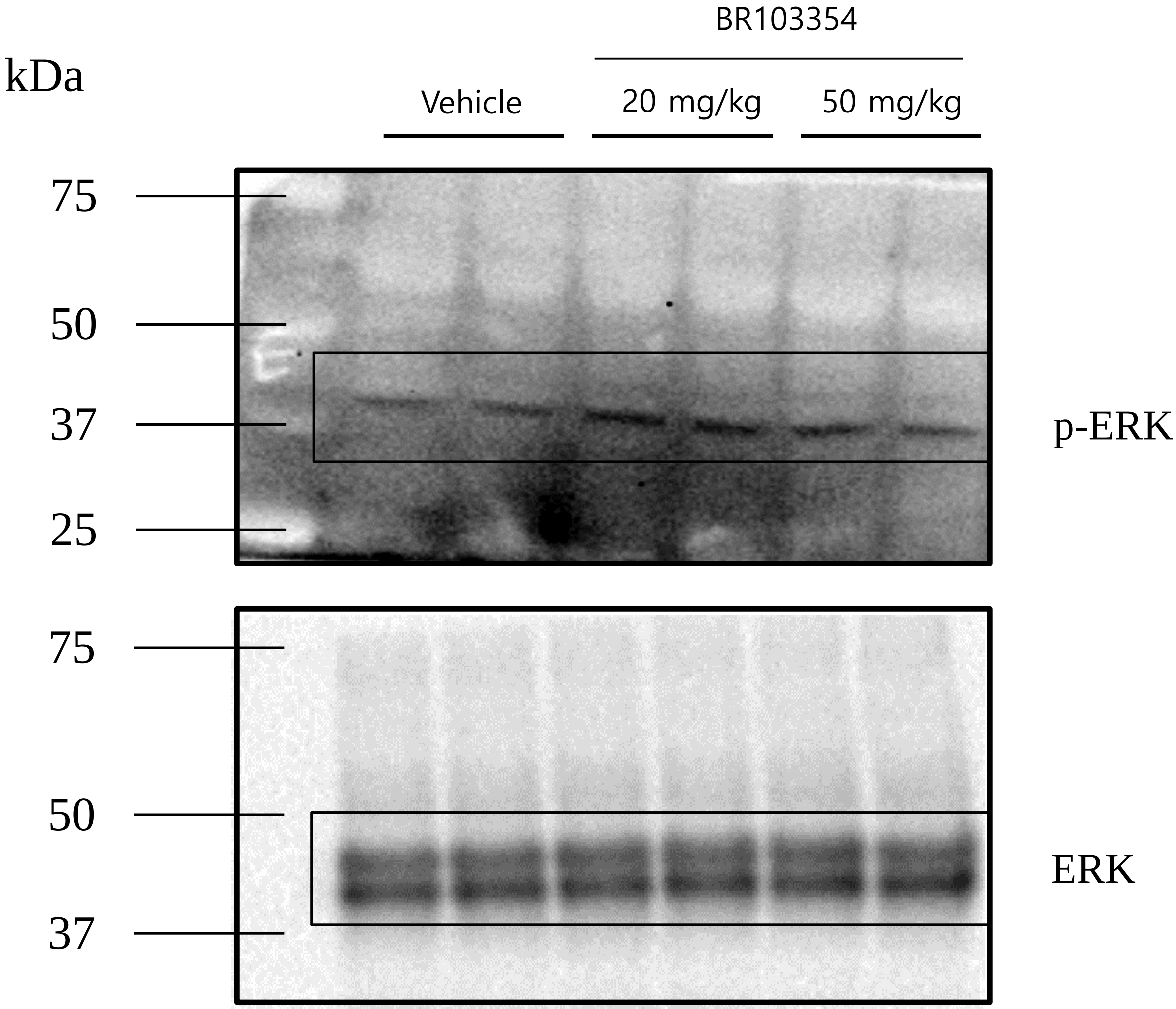

Supplementary Figure 3. Uncropped scans of western blot displayed in Fig. 4j.
